# Swallowing-related quality of life in children with oesophageal atresia: a national cohort study

**DOI:** 10.1007/s00431-022-04677-4

**Published:** 2022-11-04

**Authors:** Sandra Bergmann, Laura Antonia Ritz, Anke Widenmann-Grolig, Stephan Jechalke, Dietrich von Schweinitz, Jochen Hubertus, Eberhard Lurz

**Affiliations:** 1 Department of Paediatric Surgery, Dr. von Hauner Children’s Hospital, University Hospital, LMU Munich, Munich, Germany; 2 Sana Klinikum Lichtenberg, Berlin, Germany; 3 Patient Support Group KEKS e.V., Stuttgart, Germany; 4 Department of Paediatric Surgery, Marien Hospital Witten, University Hospital of the Ruhr University Bochum, Bochum, Germany; 5 Department of Paediatrics, Dr. von Hauner Children’s Hospital, University Hospital, LMU Munich, Munich, Germany

**Keywords:** Oesophageal atresia (OA), Swallowing quality (SWAL-QoL), Very low birth weight (VLBW), Extreme low birth weight (ELBW), Oesophageal repair

## Abstract

Swallowing and feeding disorders are a major concern for children with oesophageal atresia (OA) after primary or staged OA repair. Primary OA repair is associated with higher rates of short-term complications in preterm infants with very low birth weight (VLBW) or extreme low birth weight (ELBW). On the other hand, primary repair may have the benefit of early commencement of oral feedings. We hypothesize that also in the medium-term, swallowing-related quality of life is better after primary oesophageal repair. We conducted a prospective cross-sectional study on swallowing quality in a national cohort of former VLBW and ELBW children with OA, using the structured paediatric swallowing quality of life (*pedSWAL-QOL*) questionnaire. Results were correlated with surgical approach and baseline clinical data. Principal component analysis of *pedSWAL-QOL* domains was performed. In total, 44 complete data sets of 78 children were available. The mean age of children was 8.5 years (SD = 7.4), and 23 children (52%) had primary OA repair. The overall median *pedSWAL-QOL* score was 2 (IQR = 0–3), representing a high swallowing-related quality of life, independent of surgical technique (*p* = 0.086). Children with a history of intracranial haemorrhage (ICH) (*p* = 0.002) and those with VACTERL association (*p* = 0.008) had significantly decreased enjoyment with eating. In addition, children with VACTERL association had problems to find suitable foods (*p* = 0.04).

*Conclusion*: In this national cohort of VLBW and ELBW preterm-born children with OA, swallowing-related quality of life is good, mostly independent of initial surgery. Children with OA and ICH or VACTERL association may require more intense support with feeding.

**Table:** 

**What is Known:** *• Dysphagia, resembling feeding and swallowing disorders, is common in children and adults with repaired oesophageal atresia. Nevertheless, dysphagia in children with oesophageal atresia decreases with age.* *• Parents of younger children suffer from increased anxiety and fear regarding eating and swallowing abilities of their children.*	
**What is New:** *• Swallowing-related quality of life in former preterm children with oesophageal atresia is good, independent of initial surgical approach (primary vs. staged repair), even in very low birth weight or extreme low birth weight infants. * *• Children suffering from VACTERL association or intracranial haemorrhage show decreased enjoyment with eating.*

**Supplementary Information:**

The online version contains supplementary material available at 10.1007/s00431-022-04677-4.

## Introduction

Oesophageal atresia (OA) is the most common congenital anomaly of the oesophagus, and children with OA are born with interrupted oesophageal continuity, often combined with a tracheoesophageal fistula (TEF). OA is categorized by gross (1953) into five types (A to E), depending on the presence or absence, number and location of TEF. During the last decades surgical management has improved, and overall survival rates are greater than 90% [1]. Regardless of anastomotic strictures, re-fistulas, oesophageal dysmotility, gastroesophageal reflux disease (GERD), recurrent respiratory tract infections and wheezing, eosinophilic esophagitis (EoE), dysphagia and failure to thrive are common clinical problems [2].

Primary and staged repair are both established, but complex surgical approaches for neonates with OA [3, 4]. In primary repair, ligation of a TEF and oesophageal anastomosis are performed concurrently. In primary repair direct anastomosis of the oesophagus leads to early oesophageal continuity, allows for early oral feeding and hence might improve swallowing quality [5]. In staged repair, a gastrostomy is performed during the first operation, and if present, TEF is closed, whereas oesophageal anastomosis and introduction of oral feeding are performed at a later stage [6, 7]. Several authors suggest that this approach is a valid option especially for vulnerable very low birth weight (VLBW) and extreme low birth weight (ELBW), children with OA, as it goes along with less short-term surgical complications[3, 6, 7].

Dysphagia, resembling feeding and swallowing disorders, is common in children and adults with repaired OA [8–18]. Moreover, feeding and swallowing disorders are complex and] multifactorial in nature and are related not just to the health of children but also to the well-being of their families [10, 16–20]. Underlying aetiology, comorbidities, individual growth, neuro-cognitive development and overall health can cause feeding and swallowing difficulties. They may also affect the manifestation and clinical presentation [17, 21, 22] of these difficulties. Unfortunately, several definitions for dysphagia exist with a summary of potentially associated symptoms such as feeding difficulties, coughing, choking, slow eating and stressful mealtimes, making it difficult to estimate its prevalence in children post OA repair [8, 11, 12, 16, 18, 23, 24]. Hence, the prevalence of dysphagia in infants, children, adolescents and adults with repaired OA is reported with great variability from 21 to 84% [2, 8, 10, 15, 16]. Therefore, for over 10 years, the American Speech–Language–Hearing Association prefer “feeding and swallowing disorders” as a more inclusive phrase for dysphagia, delays and disorders in eating and drinking skills and their development [19, 25]. In addition to the clinical and instrumental assessment of swallowing motor skills [21], the assessment of swallowing quality of life, using established questionnaires for caregivers or clinicians, such as the swallowing quality of life *(SWAL-QOL)* questionnaire, is of high relevance [26].

The validated *SWAL-QOL* questionnaire was first developed for adult patients in the year 2000 and then adapted for the use in children by Clayburgh et al. in 2011 [27–29]. This questionnaire does not just capture eating and drinking skills but also its very important influence on family life, making it a suitable test to assess swallowing quality of life for children post-surgical OA repair [19, 30].

Given the benefit of early commencement of oral feedings in primary OA repair, we hypothesized that primary repair goes along with higher swallowing quality of life in VLBW and ELBW children with OA. The aim of this study was to identify swallowing-related quality of life in former VLBW and ELBW preterm children with repaired OA, using the *pedSWAL-QOL*.

## Methods

Data was collected from the German patient support organization for patients with diseases of the oesophagus (KEKS e.V.). First, structured questionnaires were sent to patient families of former premature infants with OA and VLBW (birth weight < 1500 g) or ELBW (≤ 1000 g). The responses were anonymized by KEKS e.V. and provided back to our institution for analysis. The questionnaires included demographic details (gender and date of birth), clinical features (birth weight, type of OA, long-gap, congenital heart disease, heart function, intracranial haemorrhage (ICH), congenital anomalies, associated syndromes), questions on clinical/surgical management (pre-surgical ventilation, time of first operation, primary or staged repair, gastrostomy, jejunostomy, cervical oesophagostomy, weight at staged anastomosis, age at staged anastomosis) and 10 questions on outcome after surgery (post-surgical ventilation, number of operations in the first 6 months of life, anastomotic leak, recurrent fistulas, number of dilations, gastroesophageal reflux, medical treatment, fundoplication) (see Table 1 and Supplemental Table 1). To assess symptoms of dysphagia and swallowing associated quality of life, the *pedSWAL-QOL* was used (see Supplemental Table 2). The adapted version for use with paediatric patients from Clayburgh et al. [30] was translated into German (Supplemental Table 3). Unlike the original questionnaire with Likert scales ranging from 0 to 100, a scale from 0 to 10 points was used (see Supplemental Tables 2 and 3). Each response was equally weighted. An overall outcome score was built by the sum of responses. Additionally, questions were clustered into 8 domains (see Table 2), as suggested by Clayburgh et al. [30]. The responses of each subgroup were summed to an overall outcome score. For statistical analysis, the overall outcome score of the *pedSWAL-QOL* and the outcome scores of the 8 domains were clustered into severity groups (see Table 1). All patients born with OA and VLBW or ELBW were included. Exclusion criteria were birth weight > 1500 g and/or incomplete dataset.

**Table 1:** Epidemiologic data regarding chosen items of medical questionnaire and *pedSWAL-QOL* in relation to primary and staged repair

**Parameter**	***n*** (all)*	**Primary repair** *n* **= 23**	**Staged repair** ***n*** **= 21**	***p*** value** **regarding severity of dysphagia SWAL-QOL**
Severity of SWAL-QUOL				0.086 (Op-technique)
No problems (Likert 0–2)	31	19	12	
Problems (Likert 3–7)	10	4	6	
Severe problems (Likert 8–10)	3	0	3	
Gender				0.877
Male	20	8	12	
Female	24	15	9	
Birth weight (mean)	1209 g (SD = 232)	1228 g (SD = 228)	1188 g (SD = 240)	
Birth weight				0.553
VLBW (> 1000– ≤ 1500 g)	34	19	15	
ELBW (≤ 1000 g)	10	4	6	
Type of EA (gross)				
Type A	6	0	6	
Type B	1	1	0	
Type C	36	21	15	
Unknown	1	1	0	
Time of secondary oesophageal anastomosis				
< 1 month			1	
> 1 month < 3 months			7	
> 3 months < 6 months			7	
> 6 months			3	
Unknown			3	
Weight at secondary oesophageal anastomosis				
2–2.5 kg			6	
> 2.5–3.5 kg			11	
Unknown			4	
Time of fistula closure				
No fistula	6	0	6	
< 24 h	10	7	3	
> 24 h < 48 h	7	5	2	
> 48 h < 5 days	8	4	4	
> 5 days < 7 days	2	1	1	
> 7 days	8	5	3	
Unknown	3	1	2	
Recurrent fistula/fistula relapse				
Yes	6	5	1	
No	27	12	15	
Unknown	11	6	5	
Age at questionnaire (mean)	8.5 years (SD = 7.4)	9.4 years (SD = 8.2)	7.1 years (SD = 6.1)	
Age group				0.374
< 5 years	20	10	10	**0.042** (fear of choking)
≥ 5 years	24	13	11	
Number of dilations				0.634
1. None	11	5	6	
2. Less than 3 or 3 dilations (within the first 2 years after surgery)	8	4	4	
3. More than 3 dilations( within the first 2 years after surgery)	19	10	9	
4. Less than 3 or 3 dilations (after the 2nd birthday)	1	1	0	
5. More than 3 dilations (after the 2nd birthday)	2	2	0	
Ventilations days				0.679
≤ 7 days	10	5	5	
8–30 days	20	14	6	
≥ 31 days	9	3	6	
Unknown	5			
Congenital anomalies				0.461
Yes	19	11	8	
No	24	11	13	
Unknown	1	1	0	
VACTERL				0.348
Yes	10	5	5	**0.039** (selection of food)
No	33	17	16	**0.008** (desire, enjoyableness resp.)
Congenital syndromes				
Yes	1	0	1	
No	43	23	21	
ICH				0.459
Yes	11	7	4	**0.002** (desire, enjoyableness resp.)
No	32	15	17	
Unknown	1			

Statistically significant *p* values are marked in bold

*Shows the number (*n*) of complete data sets for each item, median (IQR) and/or mean (SD). Statistical correlation of primary/staged repair and each category (e.g. severity of *pedSWAL-QOL*, birth weight, age) was tested

**The *p* value relates to the category, not the single parameter in a category, and is calculated without unknown cases performing chi-square test

**Table 2:** Domains of adapted paediatric swallowing quality of life questionnaire (*pedSWAL-QOL*), direction of the questions and their single item content. The used terms are in bold

**Cluster**	**Directed at**	**Items (shortform)**
**Swallowing-related burden**	Parents/caregiver	Dealing with child’s swallowing problem; child’s swallowing problem as major distraction in family’s life
**Duration of meals**	Child	Takes longer to eat; takes forever to eat a meal
**Desire** (enjoyableness of eating)	Child	Does not enjoy eating anymore; loses interest in eating
**Selection of food**	Parents/caregiver	Difficult to find foods; figuring out what the child can eat as family problem
**Fear of choking**	Parents/caregiver	Fear choking when eating solid food; worried about child getting pneumonias; never knowing when child is going to choke
Mental health (**level of stress**)	Parents/caregiver	Having to be so careful is annoying; discouraged by child’s swallowing problem; frustrated by child’s swallowing problem; getting impatient dealing with child’s swallowing problem
**Social function** (swallowing-related restrictions within family life)	Child/family	Swallowing problem makes it difficult to socialize with other children; families’ usual activities changed because of child’s swallowing problem; social gatherings less enjoyable because of child’s swallowing problem; eating outside difficult because of the child’s swallowing problem
**Symptoms of dysphagia**	Child	Coughing; choking on solid food; choking on liquids; thick saliva or phlegm; excess saliva or phlegm; gagging; chewing problems; having to clear the throat; food sticking in the throat; food sticking in the mouth; food or liquid dribbling out of the mouth; food or liquid coming out of the nose; coughing out food or liquid of the mouth when it gets stuck

Written informed consent was obtained from all participants caregivers. The study protocol was approved by the Institutional Research Ethics Board. The study was approved by the ethics committee of the Ludwig-Maximilian’s University Munich, Germany (Reference number 18–585). Statistical analysis was performed in collaboration with the Institute for Medical Information Processing, Biometry, and Epidemiology of the Ludwig-Maximilian’s University, Munich Germany using SPSS (IBM® SPSS Statistics, version 26).

Descriptive statistics were performed using Chi-square test for categorical data. Continuous variables were expressed as mean and standard deviation (SD) or median with interquartile range (IQR) as indicated. Linear univariate regression analysis and odds ratios were calculated for associations of clinical variables, surgical technique and *pedSWAL-QOL* scores. Principal component analysis was performed to identify clusters of *pedSWAL-QOL* subdomains. A *p* value of < 0.05 was considered as statistically significant.

## Results

Questionnaires were sent to 78 patients and their parents or caregivers. The return rate was almost two-thirds (*n* = 50). Six incomplete datasets were excluded from analysis. Full epidemiological data for 44 (56%) patients was available for analysis and is listed in Table 1.

Of all patients, 24 (54%) were female. Patients were born between August 1993 and May 2019. The mean age at questionnaire assessment was 8.5 ± 7.4 years. The mean birth weight was 1209 ± 232 g. OA gross type C was predominant with 36 cases (82%), followed by 6 patients (14%) with type A and 1 patient (2%) with type B. In one case (2%), OA type is unknown. The primary repair of OA was performed in 23 patients (52%) and staged repair in 21 patients (48%). The median postoperative mechanical ventilation time was 10 days (IQR = 7–30). No dilation of stenosis was needed in 11 patients (25%), and 9 patients (21%) needed less than three dilations, whereas 21 patients (48%) needed more than three dilations. Ten patients (23%) were diagnosed with VACTERL association; intracranial haemorrhage (ICH) was seen in 11 patients (25%) (see Table1).

Our results show that swallowing-related quality of life was high with low median (IQR) *pedSWAL-QOL* scores 2 (IQR 0–3). No swallowing disorders were claimed by 31 patients (70%). Minor swallowing problems were stated by 10 patients (23%) and severe problems by three patients (7%). Parents of younger children (< 5 years; *n* = 19) had more fear of choking (domain *Fear*; *p* = 0.042) and 21% scored problems as severe (*n* = 4). In univariate analysis, no significant correlation could be revealed between the severity of swallowing-related quality of life and epidemiological parameters (gender, birth weight, type of OA, age, number of dilations, ventilations days, VACTERL, ICH) (see Table 1). SWAL-QOL was also independent of surgical approach (*p* = 0.086) with a median *pedSWAL-QOL* of 0.5 (IQR 0–2) for children after primary repair and 2 (IQR 1–3) for children after staged repair, respectively.

Analysis of *pedSWAL-QOL* domains revealed that children with ICH (*n* = 11; *p* = 0.002) or VACTERL association (*n* = 10; *p* = 0.008) had significantly less enjoyment and interest in eating (domain *Desire/Enjoyableness*) compared to the overall group. Two (18%) patients with ICH and one patient (10%) suffering from VACTERL mentioned severe problems in this domain. Children with VACTERL were more often concerned by problems finding suitable food to eat (domain *selection*; *p* = 0.039). Half of this subgroup (*n* = 5) suffered from restrictions within *selection* and one patient was reporting severe problems finding foods that they liked and could eat.

**Fig. 1:**
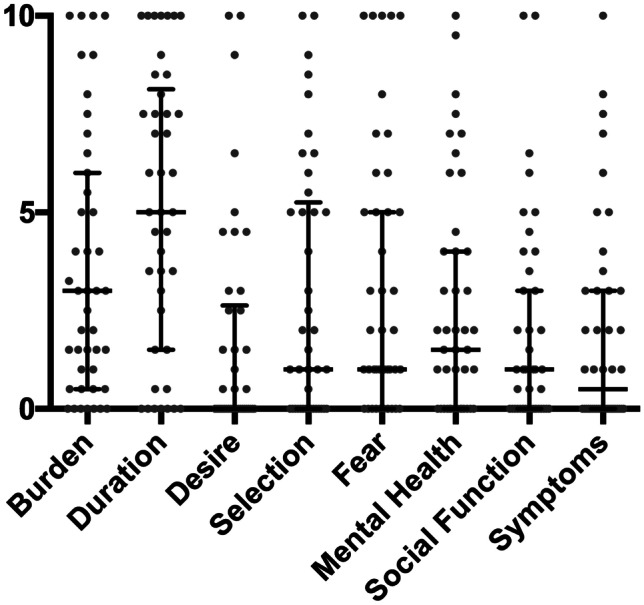
Eight subdomains (*x*-axis) of paediatric swallowing quality of life questionnaire (*pedSWAL-QOL*) and respective Likert scale results (*y*-axis)*.* Bars and whiskers display medians (interquartile range (IQR)). *Duration* was significantly higher rated as *desire/enjoyableness*, *selection of food*, *mental health/level of stress*, *social function/restrictions within family life* and s*ymptoms.* Using one-way ANOVA and Tukey’s multiple comparisons test (****p < 0,0001)

*PedSWAL-QOL* subdomains were analysed. In principal component analysis (PCA), two principal components could be detected (Bartlett test *p* = 0.000) for the entire cohort. Together, they explain 73% of result variance in *pedSWAL-QOL*. Component 1 holds 60% of variance with an eigenvalue of 4.807. This component is mainly defined by domains *level of stress* (0.860), *fear of choking* (0.851) and *swallowing-related burden* (0.824). Component 2 only explains 13% of variance (eigenvalue 1.026) mainly explained by *duration of meals* (0.876) and *desire* (0.805). Detailed results for each domain are shown in Fig. 1.

In subgroup analysis of children with primary repair, component 1 explains 55% of variance with an eigenvalue of 4.377. In patients after staged repair, a different component mostly defined by *level of stress* (0.876), *swallowing-related burden* (0.869) and *symptoms of dysphagia* (0.816) holds 60% of variance with an eigenvalue of 4.840.

## Discussion

In this national cohort of ELBW and VLBW preterm-born children with OA, we demonstrate good swallowing-related quality of life, measured by the *pedSWAL-QOL* questionnaire, independent of initial surgical approach, with 70% of children not reporting any complaints. Rather, comorbidities such as VACTERL association and ICH impact swallowing quality of life, mostly driven by *pedSWAL-QOL* domains *desire/enjoyableness* and *selection.*

Even though high rates of post-surgical complications are reported in the short-term, especially in ELBW and VLBW after primary OA repair[3, 6], in the medium-term, this does not seem to be that relevant for swallowing-related quality of life in this cohort. A high level of anxiety during mealtimes in parents of younger children was seen in our cohort where 60% of parents of infants and toddlers show significant more *fear of choking* compared to 42% parents of older children. Herewith, we confirm previous study results, where young children with OA (< 5 years) are affected by any kind of feeding or swallowing problems in about 55–70% of cases, compared to only about 18–30% of older children with OA [2, 8, 16, 31]. In a recent study from the Netherlands, self-reported health status in children with OA was also increasing with age [32]. These changes with age might be due to an accommodation of the swallowing ability and integration of feeding disorders by time [15]. As soon as children and parents cope with symptoms and adapt to altered eating abilities, they consider problems as minor or not as a problem at all, leading to a lower internal and external perception of swallowing-related problems by patients and their family members [10, 15]. Hence, the high rates of good swallowing-related quality of life in this cohort might rather resemble good adaptation to a lower level of eating skills by parents and children by time, rather than a good development in eating and swallowing skills as also suggested by Ax and colleagues[15, 31]. Courbette et al. actually did show that even though propagation of food boluses was impaired in a small number of children with OA compared to healthy controls, using high-resolution manometry, dysphagia was not related to the objective swallow assessment [33]. In fact, patients perceived swallowing quality of life might be a more important patient reported outcome (PRO) compared to objective oropharyngeal skills and perception of caregivers. Another explanation might be decreasing airway symptoms with age as reported by Dellenmark and others [34], which in general were not reported frequently in this cohort.

By using the *pedSWAL-QOL*, we were able to further analyse this concept and performed subdomain analysis. Results point in the same direction as *pedSWAL-QOL* subdomains *level of stress*, *fear of choking* and *swallowing-related burden, duration of meals* and *desire* mostly impacted the final score and not s*ymptoms of dysphagia*. Tanny et al. discuss similar experiences in their study on quality of life impact on caregivers of 100 children with OA and detected anxiety as a common phenomenon in caregivers [35]. This finding affirms Bevilaqua et al. who interviewed 51 parents of children with OA at the age of 3 years and found anxiety in 39% of cases [13]. In a retrospective audit with 75 children with OA, Menzies and Hughes further detected parental concerns about choking as a risk factor for less exposure to age-appropriate foods and textures [16]. Furthermore, in their work on impact of feeding and swallowing disorders on caregivers of 64 children with OA and TEF, Arslan et al. detected a relationship between late start of oral feeding and the risk of increased parental concerns [17]. Hence, we suggest early expert support for parents and caregivers to reduce fear and prevent children from unnecessary food restrictions. Interestingly, on the contrary, dysphagia and related symptoms such as coughing were low in this cohort, as the swallowing-specific domain *symptoms of dysphagia* had less impact on the final *pedSWAL-QOL* score. This might be related to a high proportion of OA gross type C, as more oropharyngeal motor problems are commonly seen in patients with non-type C OA, and hence, our results might be skewed towards less severe impaired eating skills[12, 20].

As a subgroup, especially children with VACTERL and ICH had poor *pedSWAL-QOL* which was heavily related to the *duration of meals* and *desire* with a remarkably high median score of 7 (IQR = 2–9) for the statement “It takes my child longer to eat than other children”. Furthermore, patients with VACTERL association were more affected by aggravated *selection of food*. VACTERL is a combination of various birth defects, such as vertebral defects, anorectal malformations, cardiac defects, OA, tracheo-oesophageal fistula and renal and limb malformations which can cause physical dysfunction. Self-perceived health status in children with OA and VACTERL is also reported to be worse compared to children with OA only [32]. VACTERL associated life-long conditions such as neurodevelopmental delay and attention deficit may lead to abnormal eating habits. These coupled with possible food bolus obstruction and intestinal dysmotility make feeding probably more complicated in children with OA and VACTERL association [32, 36, 37].Food selectivity in these children cannot only be considered as a problem but also as a coping strategy to avoid unpleasant feeding complications [24].

Children with ICH are at higher risk for neurological impairment which is strongly related to deficits in oral motor skills [16, 22, 38]. If ICH is followed by cerebral palsy, oral sensitivity and volitional oro-facial movements as well as more reflexively parts of the swallowing act may appear dysfunctional in various grades [19]. Hence, dysphagia is highly prevalent in about 66% of children with any grade of cerebral palsy [38]. Whereas dysphagia in children with OA is often related to abnormal motor function of the oesophagus [15], in children with ICH, the main problems are located in the oropharyngeal area [19, 39]. Impairment of voluntary oral movements directly affects duration of oral phase and can well explain here reported long *duration of mealtimes *[38]*.*

### Strengths and limitations of our study

Our study benefits from a homogenous cohort of VLBW and ELBW children with mainly type C OA. By using the validated and established *pedSWAL-QOL* questionnaire, a more comprehensive assessment, rather than focusing on oral feeding skills only, was possible [9]. Still, the *pedSWAL-QOL* differentiates between various eating skills and oral motor abilities [8, 19, 30] and therefore allows specific interpretations of these factors. Our study supplements further work on impact health-related quality of life of patients with OA [11, 34].

Nevertheless, our study has several limitations to consider. Due to the analysis of an anonymized data set provided by the patient support group KEKS e.V., it is neither possible to follow-up with each individual nor collect additional data on medical care. Our results therefore might be skewed as treatments with, e.g. proton pump inhibitors, or the frequency and method of oesophageal dilatations as well as the age of children at the (first) time of intervention might impact medium-term outcome of swallowing, feeding and eating. Furthermore, we have to consider a wide range regarding age at the time of survey which has an impact on the significance of our results. The poor return rate of 64%, as experienced by others assessing disease-specific health-related quality of life in children with OA [34], and additional missing data points in six more cases, led to a smaller cohort as initially targeted, and hence, our results might not be applicable to all VLBW and ELBW preterm-born infants due to potential reporting bias. Furthermore, we were not able to elaborate differences within the types of OA due to low case numbers for type A and B. Regardless of well-chosen questions, the *pedSWAL-QOL* is mainly a parent-focused tool with the risk of less or unbalanced validity from the children’s point of view. As our study was based on the analysis of a list of anonymously answered questionnaires from children with OA by the German patient support organization KEKS e.V. without outpatient or inpatient clinic visits, further clinical evaluation including growth, another significant problem in children with OA, was not possible. Direct patient approach was therewith not possible to complete missing items. Especially, correlation of *pedSWAL-QOL* results with clinical swallowing assessment tools offered by speech–language therapists, e.g. clinical swallowing assessment, videofluoroscopic swallow studies (VFSS) and fibreoptic endoscopic evaluation of swallowing (FEES), would have been interesting but could not be performed. Finally, the validated English version of *pedSWAL-QOL* was freely translated into German in a non-structured way, making it potentially less accurate.

## Conclusion

In conclusion, independent of initial surgical approach, ELBW and VLBW preterm-born children with OA achieve a good swallowing-related quality of life in the medium-term, which is probably in part due to an adaptation of children and families to their eating and swallowing habits by time. This result might alleviate stress for the patients and care takers to be able to focus more on swallowing quality than on swallowing skills. Implementing questionnaires such as the *pedSWAL-QOL* in routine follow-up of children with OA allows better understanding of the respective needs of children after OA repair and give the opportunity to perform larger prospective longitudinal long-term studies towards a more precise medical approach of children with OA.

## Supplementary Information

Below is the link to the electronic supplementary material.

Supplementary file1 (DOCX 19 KB)

Supplementary file2 (DOCX 17 KB)

Supplementary file3 (DOCX 27 KB)

## Abbreviations

OA/EA: Oesophageal/esophageal atresia
ELBW: Extreme low birth weight
EoE: Eosinophilic esophagitis
FEES: Fibreoptic endoscopic evaluation of swallowing
GERD: Gastroesophageal reflux disease
ICH: Intracranial haemorrhage
*pedSWAL-QOL*: Paediatric swallowing quality of life questionnaire
SLTs: Speech–language therapists
SWAL-QOL: Swallowing-related quality of life
TEF: Tracheoesophageal fistula
VFSS: Videofluoroscopic swallow studies
VLBW: Very low birth weight

## Statistical abbreviations

IQR: Interquartile range
*n*: Number
*p*: *p* Value
PCA: Principal component analysis
SD: Standard deviation
