# Supplementary material for: Swallowing-related quality of life in children with oesophageal atresia: a national cohort study
Source: Eur J Pediatr. 2022 Nov 4;182(1):275–83. doi: 10.1007/s00431-022-04677-4 (PMC9829586; doi:10.1007/s00431-022-04677-4)
Supplement: Supplementary file 1 — Supplementary file1 (DOCX 19 KB) [file 431_2022_4677_MOESM1_ESM.docx]

Supplemental Table 1: Questionnaire on demographic details, clinical features, clinical/surgical management and outcome of surgery.

**Allgemeine Fragen zu Patient und Operation**

| **Allgemein** | Allgemeine Informationen zu Ihrem Kind. Teile der Informationen finden sich in den Arztbriefen oder im Gelben Untersuchungsheft. Es wäre hilfreich, wenn Sie die anonymisierten Arztbriefe als Kopie anfügen könnten. | |
| --- | --- | --- |
| Geboren am | Monat: _____ Jahr: _____ | Geschlecht  weiblich   männlich |
| Geburtsgewicht | Gramm: _____ |  |
|  | | |
| **Diagnosen** | Informationen zur Diagnose finden sich in den Arztbriefen. Es wäre hilfreich, wenn Sie die anonymisierten Arztbriefe als Kopie anfügen könnten. | |
| Ösophagusatresie | Vogt  Typ I  Typ II  Typ IIIa  Typ IIIb  Typ IIIc  H-Fistel | |
|  | Long gap  ja  nein  unbekannt | |
| Herzfehler | Welcher:_______________ | Hämodynamisch relevant  ja  nein  unbekannt |
| Syndrom |  ja, welches: _________________________  nein  unbekannt | |
| Fehlbildungen |  ja, welche: _________________________  nein  unbekannt | |
| Hirnblutung |  Grad I  Grad II  Grad III  Grad IV  nein  unbekannt | |
|  |  | |
| **Operation** | Diese Informationen können Sie den Operationsberichten oder Arztbriefen entnehmen. Außerdem wäre es hilfreich, wenn Sie anonymisierte Operationsbericht und Arztbriefe als Kopie anfügen könnten. | |
| Vor der Operation beatmet |  ja  nein  unbekannt | |
| Fistelverschluss | im Alter von _____ Tagen  Gastrostoma  ja  nein  unbekannt  Jejunostoma  ja  nein  unbekannt  Collare Fistel  ja  nein  unbekannt | |
| Anastomose des Ösophagus | Zusammen mit dem primären Fistelverschluss  ja  nein  unbekannt  Sollte die Anastomose erst in einer zweiten Operation genäht worden sein  im Alter von _____ Tagen  bei einem Gewicht von _____ Gramm | |
| Operationen bis 6. Lebensmonat |  eine  zwei  drei  mehr als drei  unbekannt | |
| Beatmungsdauer | _____ Tage | |
| Komplikationen | Anastomoseninsuffizienz  ja  nein  unbekannt  Re-Fistel  ja  nein  unbekannt  Anastomosenstenose  ja  nein  unbekannt  Sonstige: _________________________ | |
| Bougierungen |  keine  weniger als drei  mehr als drei  unbekannt   vor dem 2. Lebensjahr  nach dem 2. Lebensjahr | |
| Gastroösophagealer Reflux |  symptomatisch  nicht symptomatisch  unbekannt   Säureblocker  unbekannt   Fundoplikatio im Alter von Monaten: _____  unbekannt   aktuell Refluxbeschwerden  keine Beschwerden | |
| Schluckstörungen | Siehe separaten Bogen | |
| Aktuelle Medikamente | ________________________________________  ________________________________________  ________________________________________ | |
| Aktuelles Gewicht | _____ kg | |
