# Supplementary material for: Swallowing-related quality of life in children with oesophageal atresia: a national cohort study
Source: Eur J Pediatr. 2022 Nov 4;182(1):275–83. doi: 10.1007/s00431-022-04677-4 (PMC9829586; doi:10.1007/s00431-022-04677-4)
Supplement: Supplementary file 2 — Supplementary file2 (DOCX 17 KB) [file 431_2022_4677_MOESM2_ESM.docx]

Supplemental Table 2: *pedSWAL-QOL*, English version (Clayburgh et al., 2011; adapted from McHorney et al., 2002).

| **Domain** | **Items** |
| --- | --- |
| *Burden* | Dealing with my child´s swallowing problem is very difficult |
|  | My child´s swallowing problem is a major distraction in our lives |
| *Duration* | It takes my child longer to eat than another children |
|  | It takes my child forever to eat a meal |
| *Desire* | My child doesn´t enjoy eating anymore |
|  | My child loses interest in eating due to his/her swallowing difficulty |
| *Selection* | It is difficult to find foods that my child likes and can eat |
|  | Figuring out what my child can eat is a problem in our family |
| *Fear* | I fear my child may start choking when eating solid food |
|  | I worry about my child getting pneumonia |
|  | I am afraid my child will choke when drinking liquids |
|  | I never know when my child is going to choke |
| *Mental* *Health* | Having to be so careful when my child eats and drinks annoys me |
|  | I´ve been discouraged by my child´s swallowing problem |
|  | My child´s swallowing problem frustrates me |
|  | I get impatient dealing with my child´s swallowing problem |
| *Social function* | My child´s swallowing problem makes it difficult for him/her to socialize with other children |
|  | Our family´s usual work or leisure activities have changed because of my child´s swallowing problem |
|  | Social gatherings (like holidays and get-togethers) are not enjoyable because of my child´s swallowing problem |
|  | It is difficult to eat outside of the home because of my child´s swallowing problem |
| *Symptoms* | Coughing |
|  | Choking when eating solid food |
|  | Choking when drinking liquid |
|  | Having thick saliva or phlegm |
|  | Having excess saliva or phlegm |
|  | Gagging |
|  | Drooling |
|  | Problems chewing |
|  | Having to clear his/her throat |
|  | Food sticking in his/her throat |
|  | Food sticking in his/her mouth |
|  | Food or liquid dribbling out of his/her mouth |
|  | Food or liquid coming out of his/her nose |
|  | Coughing food or liquid out of his/her mouth when it gets stuck |
