# Supplementary material for: Swallowing-related quality of life in children with oesophageal atresia: a national cohort study
Source: Eur J Pediatr. 2022 Nov 4;182(1):275–83. doi: 10.1007/s00431-022-04677-4 (PMC9829586; doi:10.1007/s00431-022-04677-4)
Supplement: Supplementary file 3 — Supplementary file3 (DOCX 27 KB) [file 431_2022_4677_MOESM3_ESM.docx]

Supplemental Table 3: *pedSWAL-QOL* in the German version. Each question is answered by Likert scale from 0 (“not at all”) up to 10 (“totally agree”).

| Es ist schwierig mit dem Schluckproblem meines Kindes umzugehen. | stimmt nicht stimmt völlig  0 – 1 – 2 – 3 – 4 – 5 – 6 – 7 – 8 – 9 – 10 |
| --- | --- |
| Das Schluckproblem unseres Kindes beeinflusst unser alltägliches Leben. | stimmt nicht stimmt völlig  0 – 1 – 2 – 3 – 4 – 5 – 6 – 7 – 8 – 9 – 10 |
| Mein Kind isst langsamer als andere Kinder | stimmt nicht stimmt völlig  0 – 1 – 2 – 3 – 4 – 5 – 6 – 7 – 8 – 9 – 10 |
| Es dauert eine Ewigkeit bis mein Kind mit einer Mahlzeit fertig ist. | stimmt nicht stimmt völlig  0 – 1 – 2 – 3 – 4 – 5 – 6 – 7 – 8 – 9 – 10 |
| Mein Kind kann das Essen nicht genießen. | stimmt nicht stimmt völlig  0 – 1 – 2 – 3 – 4 – 5 – 6 – 7 – 8 – 9 – 10 |
| Mein Kind möchte wegen des Schluckproblems nicht mehr essen. | stimmt nicht stimmt völlig  0 – 1 – 2 – 3 – 4 – 5 – 6 – 7 – 8 – 9 – 10 |
| Für mein Kind ist es schwierig Nahrungsmittel zu finden, die es gerne isst. | stimmt nicht stimmt völlig  0 – 1 – 2 – 3 – 4 – 5 – 6 – 7 – 8 – 9 – 10 |
| Es belastet unsere Familie Essen für mein Kind zu finden. | stimmt nicht stimmt völlig  0 – 1 – 2 – 3 – 4 – 5 – 6 – 7 – 8 – 9 – 10 |
| Ich habe Angst, mein Kind könnte beim Essen fester Speisen ersticken. | stimmt nicht stimmt völlig  0 – 1 – 2 – 3 – 4 – 5 – 6 – 7 – 8 – 9 – 10 |
| Ich habe Angst davor, dass mein Kind Lungenentzündungen bekommt | stimmt nicht stimmt völlig  0 – 1 – 2 – 3 – 4 – 5 – 6 – 7 – 8 – 9 – 10 |
| Ich habe Angst, mein Kind könnte jederzeit ersticken. | stimmt nicht stimmt völlig  0 – 1 – 2 – 3 – 4 – 5 – 6 – 7 – 8 – 9 – 10 |
| Es ist sehr anstrengend, das Essverhalten meines Kindes so streng beobachten zu müssen. | stimmt nicht stimmt völlig  0 – 1 – 2 – 3 – 4 – 5 – 6 – 7 – 8 – 9 – 10 |
| Ich bin wegen der Schluckprobleme meines Kindes frustriert. | stimmt nicht stimmt völlig  0 – 1 – 2 – 3 – 4 – 5 – 6 – 7 – 8 – 9 – 10 |
| Ich fühle mich wegen der Schluckprobleme meines Kindes entmutigt. | stimmt nicht stimmt völlig  0 – 1 – 2 – 3 – 4 – 5 – 6 – 7 – 8 – 9 – 10 |
| Ich werde ungeduldig, wenn ich mich um die Schluckprobleme meines Kindes kümmern muss. | stimmt nicht stimmt völlig  0 – 1 – 2 – 3 – 4 – 5 – 6 – 7 – 8 – 9 – 10 |
| Unsere Familienfreizeit Aktivitäten haben sich wegen der Schluckprobleme unseres Kindes geändert. | stimmt nicht stimmt völlig  0 – 1 – 2 – 3 – 4 – 5 – 6 – 7 – 8 – 9 – 10 |
| Große Feste mit der Familie oder Freunden sind aufgrund der Schluckprobleme unseres Kindes schwierig. | stimmt nicht stimmt völlig  0 – 1 – 2 – 3 – 4 – 5 – 6 – 7 – 8 – 9 – 10 |
| Es ist schwierig mit meinem Kind wegen der Schluckprobleme auswärts essen zu gehen. | stimmt nicht stimmt völlig  0 – 1 – 2 – 3 – 4 – 5 – 6 – 7 – 8 – 9 – 10 |
| Mein Kind hustet. | stimmt nicht stimmt völlig  0 – 1 – 2 – 3 – 4 – 5 – 6 – 7 – 8 – 9 – 10 |
| Mein Kind würgt/verschluckt sich wenn es feste Nahrung isst. | stimmt nicht stimmt völlig  0 – 1 – 2 – 3 – 4 – 5 – 6 – 7 – 8 – 9 – 10 |
| Mein Kind würgt/verschluckt sich wenn es trinkt. | stimmt nicht stimmt völlig  0 – 1 – 2 – 3 – 4 – 5 – 6 – 7 – 8 – 9 – 10 |
| Mein Kind hat zähen Speichel oder eitriges Sekret. | stimmt nicht stimmt völlig  0 – 1 – 2 – 3 – 4 – 5 – 6 – 7 – 8 – 9 – 10 |
| Mein Kind hat viel Speichel oder eitriges Sekret. | stimmt nicht stimmt völlig  0 – 1 – 2 – 3 – 4 – 5 – 6 – 7 – 8 – 9 – 10 |
| Mein Kind muss würgen. | stimmt nicht stimmt völlig  0 – 1 – 2 – 3 – 4 – 5 – 6 – 7 – 8 – 9 – 10 |
| Mein Kind hat Schwierigkeiten beim Kauen. | stimmt nicht stimmt völlig  0 – 1 – 2 – 3 – 4 – 5 – 6 – 7 – 8 – 9 – 10 |
| Mein Kind muss sich häufig räuspern. | stimmt nicht stimmt völlig  0 – 1 – 2 – 3 – 4 – 5 – 6 – 7 – 8 – 9 – 10 |
| Mein Kind hat Steckenbleiber im Hals. | stimmt nicht stimmt völlig  0 – 1 – 2 – 3 – 4 – 5 – 6 – 7 – 8 – 9 – 10 |
| Mein Kind hat Steckenbleiber im Mund. | stimmt nicht stimmt völlig  0 – 1 – 2 – 3 – 4 – 5 – 6 – 7 – 8 – 9 – 10 |
| Mein Kind speichelt Nahrungsmittel oder Flüssigkeit. | stimmt nicht stimmt völlig  0 – 1 – 2 – 3 – 4 – 5 – 6 – 7 – 8 – 9 – 10 |
| Bei meinem Kind kommt manchmal Nahrung oder Flüssigkeit aus der Nase. | stimmt nicht stimmt völlig  0 – 1 – 2 – 3 – 4 – 5 – 6 – 7 – 8 – 9 – 10 |
| Mein Kind hustet Nahrungsmittel oder Flüssigkeit / räuspert sich wenn sie im Hals stecken bleiben. | stimmt nicht stimmt völlig  0 – 1 – 2 – 3 – 4 – 5 – 6 – 7 – 8 – 9 – 10 |
| Die Schluckbeschwerden machen die soziale Interaktion schwierig für mein Kind. | stimmt nicht stimmt völlig  0 – 1 – 2 – 3 – 4 – 5 – 6 – 7 – 8 – 9 – 10 |
